# Enterovirus Transmission by Secretory Autophagy

**DOI:** 10.3390/v10030139

**Published:** 2018-03-20

**Authors:** Yael Mutsafi, Nihal Altan-Bonnet

**Affiliations:** Laboratory of Host-Pathogen Dynamics, National Heart Lung and Blood Institute, National Institutes of Health, Bethesda, MD 20892, USA; yael.mutsafibenhalevy@nih.gov

**Keywords:** secretory autophagy, picornavirus, enterovirus, poliovirus, Coxsackievirus, rhinovirus, syntaxin 17, viral transmission, quasispecies, RNA virus

## Abstract

Present in many cell types, non-degradative secretory autophagy is a newly discovered pathway in which autophagosomes fuse with the plasma membrane instead of lysosomes. Surprisingly, some viruses exploit secretory autophagy to exit cells non-lytically, shedding into the extracellular environment as particle populations contained within vesicles. As a result, this significantly enhances the infectivity of these viruses. In this paper, this novel cellular exit pathway is highlighted and its advantages for viral transmission discussed.

## 1. Introduction

The cellular process of autophagy or “self-eating” is largely considered a degradative pathway whereby organelles and cytoplasmic components are captured within de novo generated large double-membranous vesicles (DMV), so-called autophagosomes, and targeted to lysosomes, where the end products are recycled [1]. It is a pathway often induced by conditions of cellular stress, such as nutrient starvation, organelle damage, and pathogen infection. However, autophagy can also take place during normal development and differentiation, including spore formation in yeast, organelle elimination in reticulocytes, lymphocytes, and adipocytes as well as for housekeeping in terminally differentiated cells, such as neurons and hepatocytes [2].

The autophagy process is highly regulated and in mammalian cells initiated by the ULK1/2 complex. Under non-starvation/non-stress conditions, ULK1/2 is kept inactive by the serine/threonine kinase, a mechanistic target of rapamycin (mTOR) and a critical sensor and regulator of cellular homeostasis [3]. However, when cells are starved or stressed, mTOR becomes inhibited, leading to the activation of ULK1/2 and triggering autophagy. ULK1/2 regulates the production and localization of Atg9 positive vesicles, which can be derived from the endoplasmic reticulum (ER), the Golgi apparatus, and even the plasma membrane [4,5,6]. These vesicles come together and fuse to form an elongated, membranous, crescent structure called a phagophore. The subsequent ULK1/2 phosphorylation and activation of the PI3 kinase complex Beclin-1 leads to PI3P lipid generation at the phagophore which in turn recruits effectors that together work to elongate the phagophore [7]. During elongation, cargo is captured through either non-selective or selective processes. The closure of the phagophore to form the autophagosome requires the activities of two ubiquitin-like conjugation systems: Atg12-Atg5-Atg16L1 and the Atg8/LC3 family. Together, they catalyze the conjugation of phosphatidylethanolamine (PE) lipids to the Atg8/LC3 family members to generate a lipidated LC3 known as LC3-II, which remains on the autophagosome membranes while the Atg12-Atg5-Atg16L1 complex departs [8,9,10]. The critical, final steps of the degradative autophagy pathway target the autophagosome to the lysosome for fusion. This stage is poorly understood but is known to require the SNARE (soluble *N*-ethylmaleimide-sensitive factor activating receptor) proteins syntaxin17, small guanosine triphosphatase IRGM, SNAP29, VAMP8 and appears to be regulated by Atg14 [11,12,13,14].

## 2. Secretory Autophagosomes Help Viruses Get Out of Cells

Secretory autophagy is a non-degradative pathway where autophagosomes fuse with the plasma membrane and release single membrane-bound vesicles carrying cytoplasmic cargo to the extracellular environment [15]. It is widely believed to coexist with degradative autophagy in many cell types. In part due to this reason, this pathway has been difficult to study and very few of its cargo have been identified. So far, bona fide cargo includes acyl-CoA binding protein in yeast and *Dictysotelium*; mitochondria in reticulocytes during maturation; and lysozyme in Paneth cells of the intestine [16,17,18,19,20,21,22]. Notably IL-1β, which was long held as a model cargo for this pathway and exploited to identify secretory autophagy regulatory machinery such as TRIM16/sec22b/galectin-8, Rab8a, syntaxin 3 and syntaxin 4 [15,23,24] appears to also release from cells through another pathway: plasma membrane pores generated by Gasdermin D [25]. Clearly, further investigations are necessary to resolve how both pathways are utilized by IL-1β for exit. 

**Figure 1:**
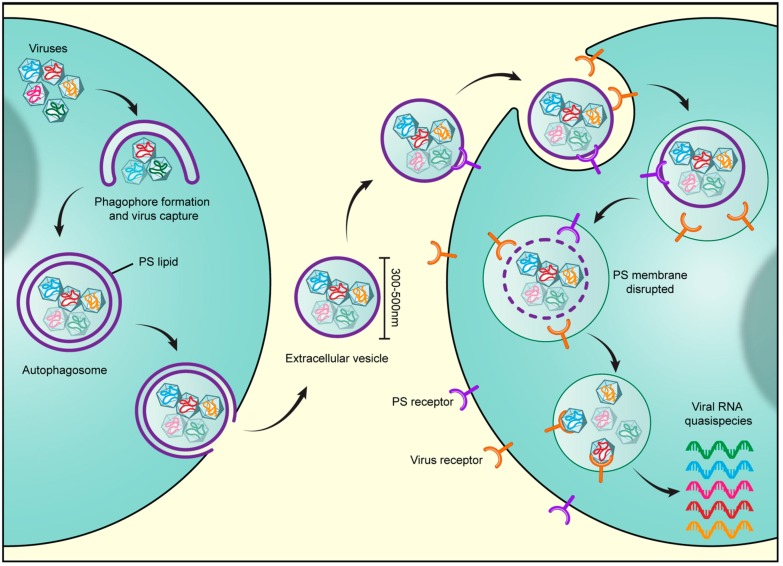
Viruses harness secretory autophagy for increased infectivity. Viruses, including the enteroviruses poliovirus, Coxsackievirus, and rhinovirus as well as rice gall dwarf virus and infectious Bursal Disease virus, are released from cells through the secretory autophagy pathway. Multiple viral particles are simultaneously captured in double-membraned autophagosomes and trafficked to the cell periphery, where the outer membrane of the autophagosome fuses with the plasma membrane. This results in the release of a unilamellar extracellular vesicle of typical size 300–500 nm, containing multiple viral particles. Notably at least for enteroviruses, the autophagosome membranes are enriched in phosphatidylserine (PS) lipids and the extracellular vesicle derived from these autophagosomes also contains PS lipids on its outer membrane leaflet. The PS lipids likely serve to dock the vesicles through interactions with PS-receptors on the receiving host cell surface. In addition, PS lipids are potent anti-inflammatory molecules. Once docked, the vesicles are internalized through endocytic pathways. Once inside endosomes, lipases likely disrupt the PS membrane and enable the viral particles to bind their cognate receptors. Viral binding to receptors results in simultaneous transfer into the host cytoplasm of multiple viral genomes. This en masse infection results in greater replication efficiency as opposed to infection by one or few viral particles as it not only enables a high multiplicity of infection but also potentially provides the opportunity for cooperative interactions to take place among viral quasispecies.

On the other hand, multiple independent studies recently have revealed that enteroviruses, positive-sense RNA viruses that include poliovirus, Coxsackievirus, enterovirus 71, and rhinovirus, exploit the secretory autophagy pathway to exit cells [26,27,28,29] (Figure 1). Enteroviruses, like all positive-sense RNA viruses, rely upon intracellular membranes for replication. Upon entry into the host cytoplasm, enteroviral RNA molecules are translated by host machinery into structural and non-structural viral proteins. The latter which include the RNA-dependent RNA polymerase and its accessory factors are assembled into a supramolecular complex on cellular membranes to synthesize viral RNA. While these membranes originate from the endoplasmic reticulum and Golgi apparatus, all enteroviruses remodel them to enrich for phosphatidylinositol 4-phosphate (PI4P) and cholesterol. Together, these two lipids facilitate viral RNA synthesis with the PI4P playing multiple roles, among them serving as a dock for viral RNA polymerases, and the cholesterol helping organize the highly negative charged PI4P lipids into domains [30,31,32]. While the PI4P is generated at these membranes by viral recruitment and the activation of a host lipid kinase, the type III phosphatidylinositol 4 kinaseβ (PI4KIIIβ), the cholesterol is brought there by viral redirection of plasma membrane cholesterol pools through the endocytic pathway [30,31].

Enterovirus release from cells was largely thought to take place through cell lysis as enteroviruses are non-enveloped. However, early electron micrographs of poliovirus-infected cells often revealed the virus particles captured within DMVs and investigations into poliovirus release from cultured intestinal epithelial cells showed polarized release [33,34]. The first indication that autophagy could be involved in the enterovirus release pathway was in a study by Jackson and Kirkegaard who showed that although autophagy was stimulated in poliovirus-infected cells, inhibiting autophagy decreased the amount of virus released from cells [29]. Note that while the overall shutdown of host protein synthesis facilitated by enteroviral factors [35] is likely a trigger for the stimulation of autophagy within infected cells, a more targeted viral mechanism cannot be excluded at this time. 

Subsequent studies by our group and others demonstrated that the bulk of poliovirus release from cells took place while the plasma membrane remained intact [26,27,28]. Furthermore, visualizing the newly assembled poliovirus nucleocapsids revealed the virus to be encapsulated within LC3-II positive autophagosomes during the time of release [26]. Surprisingly, these virus-containing autophagosomes did not meet up and fuse with lysosomes, were devoid of lysosomal enzymes throughout infection, and did not contain the SNARE protein syntaxin 17 which was instead relocated to the replication organelle membranes [26]. Significantly, the LC3-II positive autophagosomes carrying poliovirus were found to traffic to the cell periphery where the outer membrane of the DMVs fused with the plasma membrane. This resulted in the release to the extracellular environment of unilamellar vesicles (i.e., the inner membrane of the DMVs), filled with poliovirus [26]. The use of this secretory autophagy-mediated cellular exit by other enteroviruses, including Coxsackievirus and human rhinovirus, was also observed [26,28].

The secretory autophagosomes utilized by enteroviruses share some common machinery with canonical degradative autophagosomes, including Atg12, LC3, and Beclin-1, as depletion of any one of these proteins results in marked decrease in virus release [26,29]. Notably, the autophagosome membranes carrying poliovirus particles are positive for integral membrane endoplasmic reticulum (ER) proteins such as calnexin suggesting these membranes originate from the ER or the ER-derived replication organelles [26]. In addition, the timing of autophagosome biogenesis and the capture of viral particles correlates with a plateau in enteroviral RNA synthesis suggesting a transition from replication mode to release mode in the viral lifecycle [26]. As the viruses are encapsidated at the ER-derived replication organelle platforms [26], being also encapsulated within autophagosomal membranes at these sites would be viraly advantageous as it would abolish the need to traffic elsewhere and be potentially exposed en route to intrinsic host defenses.

The secretory autophagosomes carrying the enteroviruses and the extracellular vesicles derived from them are enriched in phosphatidylserine (PS) lipids. Notably, the secretory autophagosomes shuttling mitochondria out of reticulocytes were also found to be enriched in PS lipids [18,26]. The cellular source for PS lipids on these secretory autophagosomes is unclear. Although PS is synthesized de novo within the inner leaflet of the ER membrane [36,37], it is shuttled to the plasma membrane inner leaflet in exchange for PI4P [38,39]. Thus, the ER-derived autophagosomes carrying enteroviruses would not be assumed a priori to be enriched in PS lipids. However, as PI4P is generated in large quantities at the ER-derived enterovirus replication organelle membranes by the viral recruitment and activation of PI4KIIIβ [30], the typical PS/PI4P exchange, may be perturbed. As a result, PS may accumulate within the ER membranes. Alternatively, PS lipids in the inner leaflet of the plasma membrane could be routed back to the replication sites along with the cholesterol [31]. Further investigations utilizing live-cell microscopy methodologies in conjunction with fluorescent PS reporters such as Lacthedrin C2 [36] may be able to shed light on PS dynamics in the context of secretory autophagosome biogenesis both within enterovirus- infected and non-infected cells (e.g. reticulocytes).

Since these original findings on enteroviruses, secretory autophagy has also been implicated in the transmission of several other viruses with varying degrees of evidence. In particular, both the double-stranded RNA viruses, the rice gall dwarf virus (RGDV) and the infectious Bursal Disease virus (IBDV), appear to exploit this pathway to release non-lytically and spread to other cells. Upon infection with RGDV or IBDV, the host autophagy machinery including Ulk1, Atg5, and Atg8/LC3 has been reported to be upregulated similarly to enteroviruses, and modulating autophagy with inhibitors or stimulators results in expected outcomes on RGDV and IBDV release [40,41]. Furthermore, much like with enteroviruses, ultrastructural examination of infected cells has revealed RGDV and IBDV nucleocapsids to be encapsulated within LC3-II positive cytoplasmic DMVs and released to the extracellular side within unilamellar vesicles [17,40].

Recent data also point to autophagy having a potentially facilitative rather than a degradative role in the Zika virus lifecycle, specifically in its vertical transmission from placental trophoblasts to fetal cells [42,43]. Inhibiting autophagy results in decreased extracellular Zika virus titers in both cultured human trophoblasts and in mice placenta in vivo [42,44]. Moreover, in cultured skin cells, Zika virus co-localizes with autophagosomes and autophagy is required for Zika replication [45]. However, whether the Zika virus is inside the autophagosomes and whether these autophagosomes track out to the plasma membrane and fuse with it remains to be determined.

## 3. Advantages of Harnessing Secretory Autophagy for Viral Transmission

The infectivity of enteroviruses carried inside vesicles is still dependent on the next host cell expressing their cognate virus receptors, such as CD155 for poliovirus or Adenovirus receptor (CAR) for Coxsackievirus B3 [26]. This indicates that vesicles do not simply fuse with their target cells to deliver their viral cargo. Rather, the vesicle membranes become disrupted which enables the virus to bind its receptor. This disruption likely takes place after the vesicles have become internalized within a cellular endocytic compartment, since outside the cell the vesicle membrane is able to protect the viral cargo against neutralizing antibodies [46]. Notably, endocytic vesicles are known to contain a variety of lipases which can potentially facilitate the disruption of the vesicle membrane [47]. 

In addition to the virus receptors, the PS lipids exposed on the vesicle surface also appear to regulate infectivity. Masking these lipids with Annexin V, a PS-binding protein, prior to adding the vesicles to cells significantly blocks infection [26]. PS lipids are recognized by PS receptors, a large family of proteins expressed by nearly all cells [48]. Thus the PS may help dock the vesicles onto cells and stimulate their uptake [49,50]. Moreover, the interaction between PS receptors on the host cell and the PS on the vesicle may play a role in regulating the tropism of the vesicles in conjunction with other lipid and protein components on the vesicle.

Viral exploitation of the secretory autophagy pathway has significant implications for viral infectivity. First, the ability to leave cells non-lytically is an important survival advantage for any virus as cell lysis is a highly inflammatory event that attracts many components of the immune system. Secondly, once outside the cell, vesicle membranes help viruses evade recognition by neutralizing antibodies [46]. Thirdly, the PS on the vesicle membranes can act to not only enhance cellular viral uptake [49,50] but can also act as an “anti-inflammatory” agent following uptake and evade triggering the immune system [51]. As evidence for this, the PS on the vesicles exporting mitochondria out of differentiating reticulocytes enable both internalization by phagocytic cells and subsequent anti-inflammatory cytokine production [51].

Finally, viral harnessing of the secretory autophagy pathway, which results in the release of large extracellular vesicles of 300–500 nm typical diameter, enables the transport of multiple viral particles en masse to another host [26,52] and the subsequent en masse transfer of multiple viral genomes into that host’s cytoplasm [26] (Figure 1). Remarkably, inoculating a culture of cells with vesicles containing enteroviruses results in much greater replication efficiency and higher viral yield than when equivalent numbers of enteroviruses have been inoculated as free independent particles into similar numbers of cells (e.g., 1000 free viral particles versus 10 vesicles with 100 viral particles per vesicle) [26]. These results seem paradoxical as independent free viral particles would be considered to be more efficient infectious units since they would be able to infect far many more cells traveling independently of one another. Instead, these results suggest that there are replication barriers when single or few viruses enter a cell; these are overcome by the en masse transfer of multiple genomes through vesicles.

In part, replication barriers may be due to individual RNA viruses having a high probability of carrying debilitating mutations. These mutations are mainly due to errors made by RNA polymerases during replication that go uncorrected. Thus, viral progeny released from an infected host cell are a mix of quasispecies [53] rather than exact copies of each other and the parental virus. While some mutations will not have any consequence, others may have profound effects on the fate of a specific progeny in the next infection cycle: changing the secondary structure of the viral RNA or disrupting the coding sequence of a critical replication enzyme. On the other hand, when multiple quasispecies are transferred en masse into a host, such as through vesicle carriers, cooperative interactions amongst quasispecies can potentially take place at the start of infection, which can complement one another’s deleterious mutations. Notably this type of cooperativity may also result in greater genetic diversity which could help promote faster drug resistance and immune evasion [54]. Additionally, independent of the advantages of cooperative interactions provided by en masse transfer of multiple viral genomes, even a single infecting virus lacking any deleterious mutations would be vulnerable to intrinsic host defenses until, through successive cycles of translation and replication, it reached sufficient levels take over the host. In contrast, the high multiplicity of infection afforded by vesicles would enable a rapid rise in viral protein and RNA levels and a more efficient host takeover and progeny generation.

In summary, harnessing secretory autophagy has multiple significant advantages for viruses, including evasion of the immune system, achieving a high multiplicity of infection, and overcoming the drawbacks of mutations among viral progeny. While secretory autophagy is also emerging as an important cellular pathway, little is known regarding its cargo selectivity and its regulation, in particular relative to the canonical degradative autophagy pathways. Given this, enteroviral infections may be perfect model systems to shed light on this novel pathway.
